# Integrating Functional Data to Prioritize Causal Variants in Statistical Fine-Mapping Studies

**DOI:** 10.1371/journal.pgen.1004722

**Published:** 2014-10-30

**Authors:** Gleb Kichaev, Wen-Yun Yang, Sara Lindstrom, Farhad Hormozdiari, Eleazar Eskin, Alkes L. Price, Peter Kraft, Bogdan Pasaniuc

**Affiliations:** 1 Bioinformatics Interdepartmental Program, University of California Los Angeles, Los Angeles, California, United States of America; 2 Department of Computer Science, University of California Los Angeles, Los Angeles, California, United States of America; 3 Program in Genetic Epidemiology and Statistical Genetics, Harvard School of Public Health, Boston, Massachusetts, United States of America; 4 Department of Human Genetics, David Geffen School of Medicine, University of California Los Angeles, Los Angeles, California, United States of America; 5 Department of Biostatistics, Harvard School of Public Health, Boston, Massachusetts, United States of America; 6 Department of Pathology and Laboratory Medicine, David Geffen School of Medicine, University of California Los Angeles, Los Angeles, California, United States of America; University of Chicago, United States of America

## Abstract

Standard statistical approaches for prioritization of variants for functional testing in fine-mapping studies either use marginal association statistics or estimate posterior probabilities for variants to be causal under simplifying assumptions. Here, we present a probabilistic framework that integrates association strength with functional genomic annotation data to improve accuracy in selecting plausible causal variants for functional validation. A key feature of our approach is that it empirically estimates the contribution of each functional annotation to the trait of interest directly from summary association statistics while allowing for multiple causal variants at any risk locus. We devise efficient algorithms that estimate the parameters of our model across all risk loci to further increase performance. Using simulations starting from the 1000 Genomes data, we find that our framework consistently outperforms the current state-of-the-art fine-mapping methods, reducing the number of variants that need to be selected to capture 90% of the causal variants from an average of 13.3 to 10.4 SNPs per locus (as compared to the next-best performing strategy). Furthermore, we introduce a cost-to-benefit optimization framework for determining the number of variants to be followed up in functional assays and assess its performance using real and simulation data. We validate our findings using a large scale meta-analysis of four blood lipids traits and find that the relative probability for causality is increased for variants in exons and transcription start sites and decreased in repressed genomic regions at the risk loci of these traits. Using these highly predictive, trait-specific functional annotations, we estimate causality probabilities across all traits and variants, reducing the size of the 90% confidence set from an average of 17.5 to 13.5 variants per locus in this data.

## Author Summary

Genome-wide association studies (GWAS) have successfully identified numerous regions in the genome that harbor genetic variants that increase risk for various complex traits and diseases. However, it is generally the case that GWAS risk variants are not themselves causally affecting the trait, but rather, are correlated to the true causal variant through linkage disequilibrium (LD). Plausible causal variants are identified in fine-mapping studies through targeted sequencing followed by prioritization of variants for functional validation. In this work, we propose methods that leverage two sources of independent information, the association strength and genomic functional location, to prioritize causal variants. We demonstrate in simulations and empirical data that our approach reduces the number of SNPs that need to be selected for follow-up to identify the true causal variants at GWAS risk loci.

## Introduction

Recent breakthroughs in high throughput genotyping technologies have ushered in the era of genome-wide association studies (GWAS) that have reproducibly identified thousands of genetic variants associated to many diseases and complex traits [1]. GWAS leverage the linkage disequilibrium (LD) patterns among genetic markers for probing genetic variation beyond the typed variants. Thus, it is often the case that the associated variant is not itself biologically causal, but rather, a proxy as a result of LD. Identification of causal variants underlying risk loci is performed within fine-mapping studies [2], [3], [4] through sequencing (or array typing and imputation) followed by variant prioritization using marginal association statistics or posterior probabilities [5], [6], [7]. Using these measures, a set of top candidate variants is selected for testing in functional experiments to validate biological causality.

Many statistical approaches have been introduced for fine-mapping ranging from a simple ranking of marginal association statistics to Bayesian approaches that integrate elaborate priors [5], [8], [9], [10], [11], . Due to the fact that fine-mapping can be casted as a variable selection problem, both LASSO-like procedures that estimate empirical probabilities of inclusion for SNPs based on sub-sampling [13], as well as Bayesian approaches that perform joint multipoint inference to compute posterior inclusion probabilities [14] have been proposed. The inclusion probabilities provided by these methods offer a natural way to prioritize variants in fine-mapping. However, although neither of the two variable selection approaches assume a fixed number of causal variants, they both require individual level data which is often not readily available. Ranking of SNPs for follow-up analysis can also be performed based on correlation-adjusted t-scores that explicitly take into account the correlation structure among variants, thus requiring individual level data [12] as well. Recent works [5], [8], [9] have proposed to estimate posterior probabilities and credible sets for variants to be causal under the simplifying assumption of single causal variant per locus. A key advantage of such approaches is that they only require marginal association statistics which are readily available for large-scale data sets.

Large-scale initiatives such as The Encyclopedia of DNA Elements (ENCODE) [18] have ascribed functional importance to more than 80% of the human genome and have provided a genome-wide catalogue of regulatory regions. This functional annotation data can be used jointly with the standard association signal to gain insights into the genetic basis of common traits. Indeed, variants associated with certain ENCODE genomic functional annotations such as DNase I Hypersensitive Sites, transcription factor binding sites and expression quantitative loci are enriched among GWAS hits [19], [20], [21], [22], [23], with recent work demonstrating that it is possible to integrate such data with the GWAS association signal to identify novel risk loci [10]. However, existing integrative frameworks typically either assume a single causal variant per risk locus [10] that is likely to be incorrect at many risk loci [10], [24], [2], [7], [25], [26], [27], [28] or do not make use of functional data [29], [30]. Although ENCODE functional annotation data are clearly beneficial for fine-mapping [22], a rigorous statistical framework for integrating the different types of information for the purpose of prioritizing plausible causal variants is currently lacking.

In this work we introduce PAINTOR (Probabilistic Annotation INTegratOR), a framework to combine external functional annotations (sets of variants that localize within certain genomic features, e.g. enhancers, repressors) with genetic association data (the strength of association between genetic variants and the phenotype) to improve the prioritization of causal variants in fine-mapping studies. As compared to existing approaches that only rely on the strength of association between genotype and phenotype [31], [5], [6], our framework combines two orthogonal lines of evidence to estimate variant-specific probabilities for causality: functional relevance and genotype-phenotype association. These probabilities can then be used for prioritization of variants for functional validation studies to determine biological causality. More specifically, we incorporate the external functional annotation data through an Empirical Bayes prior [32] with parameters inferred from targeted fine-mapping data, obviating the need to make assumptions on which tissue-specific annotation is relevant to the trait of interest. Finally, budgetary constraints will invariably restrict the number of potential variants that can be validated in functional studies. We address this issue by proposing a cost-to-benefit optimization framework to guide the design of experimental follow-up studies.

We use extensive simulations starting from the 1000 Genomes data to show that our approach improves resolution of statistical fine-mapping and is superior to existing frameworks. In our simulations of a trait with a heritability of 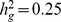 across 100 risk loci, one needs to test in functional assays an average of 12.3 SNPs per locus to identify 90% of all causal variants if using our approach. In addition, if causal variants are preferentially enriched within certain genomic regions [19], [21], [10], [23], PAINTOR further reduces the average number of SNPs per locus needed to capture 90% of the causal variants to 10.4. We show in simulations that the enrichment estimates provided by PAINTOR are largely unbiased, a fact that we can subsequently use to search for the annotations most phenotypically relevant. We then demonstrate an application of our approach using data from a large-scale meta-analysis study of blood lipid phenotypes (triglycerides (TG), total cholesterol (TC), high density lipoprotein (HDL), low density lipoprotein (LDL) [33])and find that causal variants at risk loci are preferentially enriched within coding regions and significantly depleted from repressed regions. In real data, PAINTOR is able to reduce the size of the 90% confidence set from an average 17.5 to 13.5 SNPs per locus, a reduction consistent to simulation results. We provide software implementing our framework freely available to the research community at http://bogdan.bioinformatics.ucla.edu/software/paintor/.

## Results

### Overview of statistical fine-mapping with functional annotation

**Figure 1:**
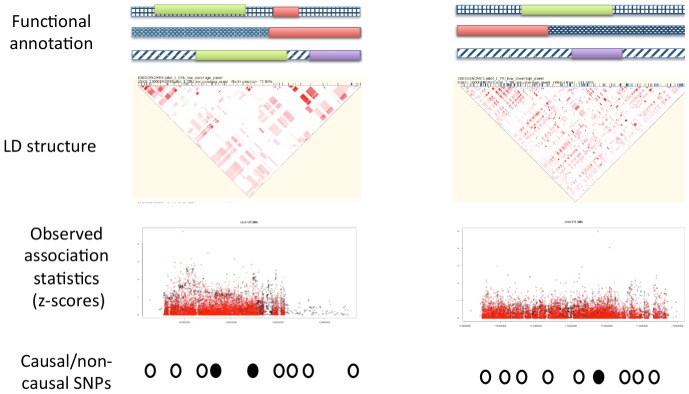
Illustration of model input. PAINTOR is a statistical model for incorporating functional annotations on top of association statistics to ascribe probabilistic confidence of causality to the SNPs at the loci. Depicted here are two loci with functional annotations from three different cell lines/tissues and three different classes. Causal variants are enriched within the green annotation class while depleted from others. PAINTOR is designed to upweight (with probability mass) SNPs residing in the green annotation while down-weighting SNPs residing in the red annotation.

To illustrate PAINTOR, consider the case of two risk loci that are fine-mapped through sequencing to elucidate the causal variant(s) driving the phenotype (Figure 1). The observed association statistics at all SNPs at these loci are a function of the causal variants, their effect size and the locus-specific LD structure. We use a multivariate normal approximation to connect the LD structure of a fine-mapping locus to the association statistics (e.g. association z-scores) which allows for the possibility of modeling multiple causal variants – an important feature since the number of causals variants per locus is typically unknown a priori. We integrate functional annotation data through an Empirical Bayes prior [32] such that the prior probability of a variant to be causal is governed by its membership to functional classes (see Methods). We perform maximum likelihood estimation over all fine-mapping loci using a variant of the Expectation Maximization algorithm to infer the parameters of the model, followed by estimation of the probabilities for each variant to be causal (see Methods). Intuitively, PAINTOR up-weights variants residing in certain functional annotations (e.g. transcription start sites) while down-weighting variants within annotations less relevant to the trait (e.g. intergenic). The weight associated to each functional annotation is inferred from the data itself without making any ad-hoc assumptions on which tissue-specific annotations are relevant to the trait of interest. The main output of PAINTOR is a probability for each variant to be causal that can be used for selection of SNPs to be tested for biological causality in functional assays.

### Functional annotation data improves statistical fine mapping performance

Numerous approaches for fine-mapping have been proposed, ranging from methods that require individual genotype data to methods that take as input summary association data and integrate functional annotations (see Table S1). We used simulations to compare PAINTOR to previously proposed methods. It is generally the case that in fine-mapping studies several risk loci are simultaneously sequenced (or densely genotyped) and a set of plausible causal SNPs is selected for follow-up in functional assays. We therefore simulated fine-mapping data sets across one hundred 10 KB risk loci that collectively explained 25% of the phenotypic variance 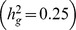 in N = 10,000 individuals. We created three synthetic “functional annotations” that roughly correspond to coding exons (2.2% of all variants), transcription start sites (2.2% of all variants), and DNase Hypersensitivity Sites (30.7% of all variants) and enriched them with causal variants at 9.5, 5.7 and 3.7-fold to approximately match what we observed in real data (see below). Each simulation resulted in approximately 64 loci that harbor at least one causal variant with 34 harboring a single causal variant and the remaining harboring multiple causal variants (see Methods). We compared all approaches across only loci with at least one causal variant.

**Figure 2:**
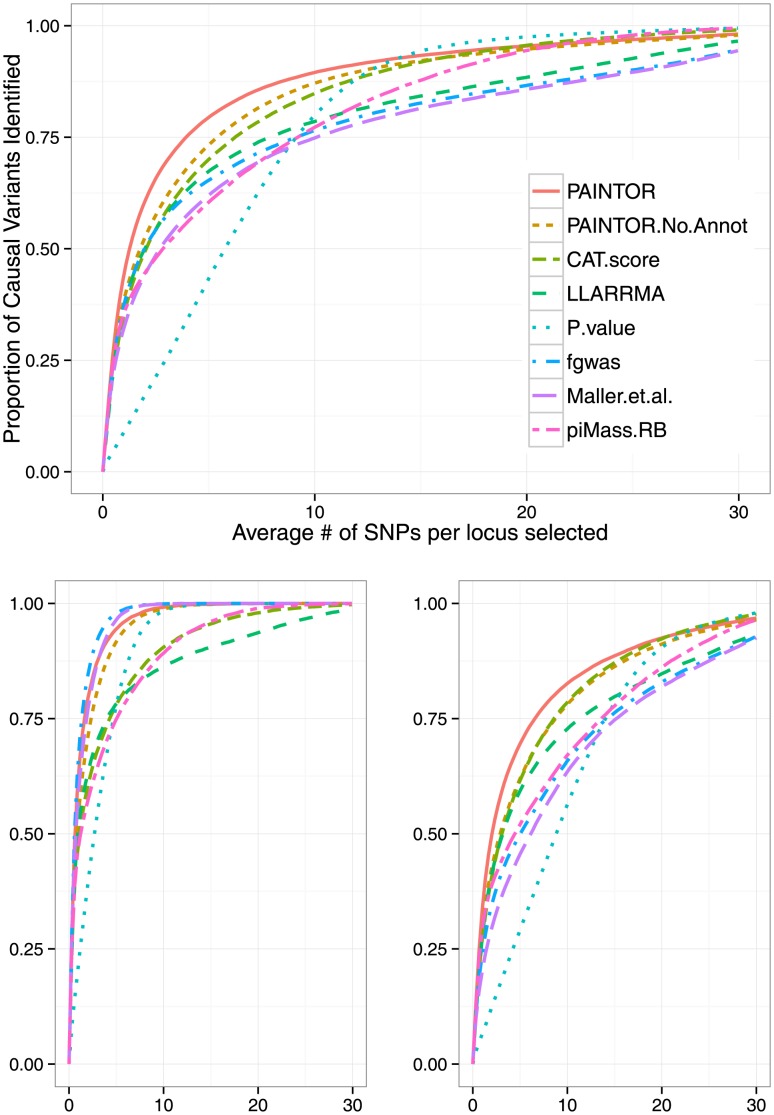
PAINTOR outperforms existing methodologies for fine-mapping. We simulated datasets consisting of 10 K genotypes over one hundred 10 KB loci using three synthetic functional annotations randomly dispersed at fixed percentages (2.2%, 2.2%, 30.7%). SNPs falling within these annotations were enriched (9.5, 5.7, 3.65) times more with causal variants relative to unannotated SNPs. We fixed the variance explained by these loci to 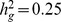 and repeated the simulation 500 times. The top figure corresponds to the overall performance at causal loci (64 loci) with PAINTOR clearly achieving the greatest overall accuracy. The bottom figures correspond to loci with a single causal variant (an average of 34 per simulation) (left) or multiple causal variants (average of 30 per simulation) (right). At loci where there is one true causal variant, fgwas achieves greater accuracy than PAINTOR due to the fact that fgwas assumes the correct number of causal variants. We note that the version of PAINTOR that assumes a single causal variant yields very similar to fgwas at loci where the truth is of a single causal (both requiring 2.63 SNPs per locus to identify 90% of the causal variants.) However, at loci with multiple causal variants, the power of methods that assume a single causal is greatly deflated leading to PAINTOR's superior overall accuracy.

We find that prioritizing variants using PAINTOR posterior probabilities achieves superior accuracy over existing methodologies (see Figure 2 and Table 1). Our approach identifies more causal variants at all selection thresholds, and is a consequence of PAINTOR's ability to model multiple causal variants while incorporating functional priors. For example, in order to find (50%, 90%) of all causal variants one needs to select an average of (1.3, 10.4) SNPs per locus if using PAINTOR. In contrast, ranking SNPs using frameworks that assume a single causal variant, such as Maller et al. [5] and fgwas [10], require (2.7, 25.4) and (2.0, 21.5) SNPs per locus, respectively. In general, we observe an increase in performance for methods that incorporate functional data and allow for multiple causal variants at a risk locus (see Tables 1 and 2). Despite having access to individual level data, variable selection strategies [13], [14] were less accurate than PAINTOR in our simulations (see Figure 2 and Table 1). Ranking SNPs based on correlation-adjusted t-scores [12] was superior to existing methodologies, however, still failed to achieve the same level of accuracy of PAINTOR, requiring an average of (2.0,13.3) SNPs per locus to find (50%, 90%) of all causal variants. Across all methodologies, the relative performance holds irrespective of whether SNPs are prioritized across all fine-mapping loci or within each locus independently (generally the latter strategy is sub-optimal (see Table 1)). Finally, we note that iterative conditioning, a method typically used to detect multiple independent signals, performs worse than the prioritization strategies described here (see Figure S1) [7]. Interestingly, as the number of SNPs selected for follow-up increases, the naive approach of selecting based on association p-value alone attains high accuracy, most likely due to the much smaller set of assumptions as compared to other methods.

**Table 1:** Summary of performance for various fine-mapping methods.

				Variant Ranking Across All Loci		Variant Ranking Independently at Each Locus	
Method	Summary Data	Incorporates Annotation	Assumed Num. of Causal Variants	Causals Identified		Causals Identified	
				50%	90%	50%	90%
P-value	Yes	No	n/a	5.74	12.60	2.94	19.15
CAT score [12]	No	No	n/a	2.04	13.29	2.56	17.80
LLARRMA [13]	No	No	Mult	1.98	21.93	2.46	23.11
piMass-RB [14]	No	No	Mult	2.83	16.31	2.18	15.15
Maller et al. [5]	Yes	No	Single	2.68	25.44	2.96	19.13
fgwas (no annot)	Yes	No	Single	2.69	25.48	2.95	19.11
PAINTOR (no annot,1CV)	Yes	No	Single	2.69	22.49	2.95	19.09
fgwas [10]	Yes	Yes	Single	1.95	24.77	2.05	17.37
PAINTOR (1CV)	Yes	Yes	Single	1.95	21.51	2.03	17.43
PAINTOR (no annot)	Yes	No	Mult	1.76	12.25	2.24	16.86
**PAINTOR**	**Yes**	**Yes**	**Mult**	**1.26**	**10.42**	**1.61**	**13.68**
PAINTOR True	Yes	Yes	Mult	1.23	10.22	1.59	13.48

Methods were benchmarked using the average number of SNPs per locus selected to find (50%,90%) of all causal variants. We simulated a trait with 100 risk loci explaining 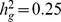 fine-mapped through sequencing of N = 10,000 samples and assessed accuracy only at loci that harbor at least one casual variant (64 loci on the average). We explored two methods to prioritizing variants: (1) “Variant Ranking Across All Loci” prioritizes SNPs across all loci while (2) “Variant Ranking Independently at Each Locus”, first prioritizes variants at each risk locus followed by merging across all loci. We note that PAINTOR 1CV and/or no annot corresponds to running PAINTOR assuming a single causal variant and/or not providing access to annotations. PAINTOR True did not empirically estimate enrichment but used the true enrichment values for each functional annotation data.

**Table 2:** Leveraging functional priors leads to improved fine-mapping resolution.

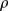 -level	Method	Annotations	Causals Identified	SNPs Selected
90%	Maller et al.	−	64.2	265.0
	fgwas	+	64.5	209.6
	PAINTOR	−	91.9	510.3
	PAINTOR	+	91.2	393.7
95%	Maller et al.	−	69.6	343.7
	fgwas	+	70.2	290.8
	PAINTOR	−	97.2	687.8
	PAINTOR	+	97.0	567.2
99%	Maller et al.	−	77.7	506.6
	fgwas	+	77.9	457.3
	PAINTOR	−	102.6	1074.4
	PAINTOR	+	102.7	954.3

We define an *ρ*-level confidence set as the number of SNPs we need to select in order to consume an 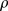 fraction of the total posterior probability mass over all loci. Results in the table correspond to averaging over 500 independent simulations where the average number of true causals SNPs per simulation was 109.2. The size of 90%, 95%, and 99% confidence sets are reduced by 22.8%, 17.5% and 11.1% when incorporating functional annotations as prior probabilities. Methods that assume one causal variant are miss-calibrated due to loci with multiple causals.

### Factors impacting fine-mapping performance

Having established that PAINTOR increases fine-mapping accuracy over existing methods in simulations, we next explored the gain in performance attributable to having access to functional annotation data. We find that prioritizing variants using PAINTOR with functional data increases accuracy at all significance thresholds. For example, in order to find (50%, 90%) of all causal variants one needs to select an average of (1.3, 10.4) SNPs per locus if integrating functional data as opposed to (1.7, 12.3) if excluding annotation data. We note that our approach that does not empirically estimate the prior, but uses the known prior information does not lead to superior performance over PAINTOR in these simulations (see Table 1) reflecting the fact that the prior probability for each SNP is accurately estimated. Furthermore, as the size of the fine-mapping locus is increased, PAINTOR continues to outperform simpler approaches. In particular, to resolve 90% of the causal variants for loci (10 Kb, 25 Kb, 50 Kb) in size, one needs to select (27.4, 52.3, 110.7) SNPS per locus if ranking on posterior probabilities assuming a single causal variant as opposed to (11.4, 16.0, 24.1) SNPs per locus if ranking using PAINTOR (see Table 3).

**Table 3:** Performance of PAINTOR compared to standard methodologies at variable sized loci.

Locus Size	%Causal	p-value	Maller et al.	PAINTOR
10 Kb	10%	1.04	0.17	0.17
	50%	5.73	2.20	1.35
	90%	12.87	27.35	11.41
25 Kb	10%	1.68	0.16	0.16
	50%	8.88	2.73	1.57
	90%	21.93	52.32	16.01
50 Kb	10%	2.50	0.17	0.16
	50%	13.69	3.65	1.87
	90%	36.92	110.69	24.07

To expedite simulations, we used a modified version of the simulation setup. As before, causal SNPs were drawn according to a logistic prior such that in expectation there were a total of 100 causal variants – we did not enrich causal in any annotations. For this experiment, Z-scores were drawn directly from a multivariate normal distribution; this gave virtually identical results to using simulated genotypes derived from HAPGEN (see Methods). We find that PAINTOR increasingly outperforms existing methodologies as the size of the loci become larger.

We next sought to determine at what types of loci is functional prior data providing the biggest increase in accuracy. Loci where the association signal is strong (i.e. loci where the p-value at the causal variants are in the top quartile across all loci with at least one causal variant) do not gain much from integration of functional annotation data, with the number of SNPs required to find 90% of the causal variants decreasing by only 6.5%. On the other hand, at loci where the association signal is weak (i.e. loci where the p-value at the causal variants are in the bottom quartile) we observe a 21.4% decrease in the total number of SNPs to be followed-up to find 90% of all causal variants (see Table S2). This suggests that as the causal status for a SNP becomes increasingly ambiguous on the basis of association data alone (e.g. small effect size), the importance of incorporating additional sources of information is magnified.

It is not guaranteed that the true causal variant will be present in the fine-mapping data set due to technical reasons (e.g. capture sequencing technology or imputation accuracy). To explore this scenario, we simulated fine-mapping data sets at a locus 100 Kb in size after which we masked the true causal(s) from the data (see Methods). To measure fine-mapping performance when causal variant is absent from the data, we looked at the distance in base-pairs between variants in the top N SNPs to the true masked causal SNP. As expected, we observed a decrease in performance when causal variants are absent from the fine-mapping dataset (e.g. the average median distance to the true causal variant in the set of top 5 SNPs increases by 6% when the causal variant is masked, see Table 4). The rather small nominal decrease in localization distance suggests that accurate localization may be attained even in the absence of the causal variant.

**Table 4:** Fine-mapping resolution when causal variant is missing.

Method	Causal Variants	N = 1	N = 5	N = 10	N = 25
PAINTOR	Typed	17.6 (17.6)	20.4 (3.2)	21.6 (1.6)	23.1(0.5)
	Masked	22.7 (22.7)	21.7(6.9)	22.0 (4.0)	23.3 (1.8)
Random	Typed	32.1 (32.1)	30.2 (8.9)	30.3 (4.5)	30.1 (1.7)
	Masked	32.0 (32.0)	30.6 (9.1)	30.6 (4.7)	30.2 (1.9)

Average median (minimum) distance in Kb to true causal variant(s) for SNPs in the set of top N SNPs when causal variant is either present or absent from the fine-mapping data set. We simulated one 100 Kb locus with causal status drawn from a uniform prior. We then masked the causal variant(s) to explore how this would effect fine-mapping resolution.

Alternatively, we can recast the observed improvement in causal variant localization when incorporating functional annotations as a decrease in size of the set of SNPs to account for a fixed amount of posterior probability mass. We extend existing work for single-locus fine-mapping [5], [8], [9], [7] to define an *ρ*-level causal set as the set of top SNPs (rank-ordered based on probabilities) across all fine-mapping loci that consume an 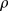 fraction of the *total* posterior probability mass. We observe a reduction in the number of SNPs within the 90%, 95% and 99% confidence sets when using functional annotations as compared to no functional data (see Table 2). In addition, although PAINTOR with annotation yields fewer SNPs with high probability than the PAINTOR with no annotation (232.8 vs 265.2 at a threshold of 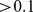), having access to annotation yields more simulated causals with high posterior probability (78.6 vs 73.8 at a threshold of 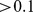) (see Table S3).

### Estimation of relevant annotation data for fine mapping

**Figure 3:**
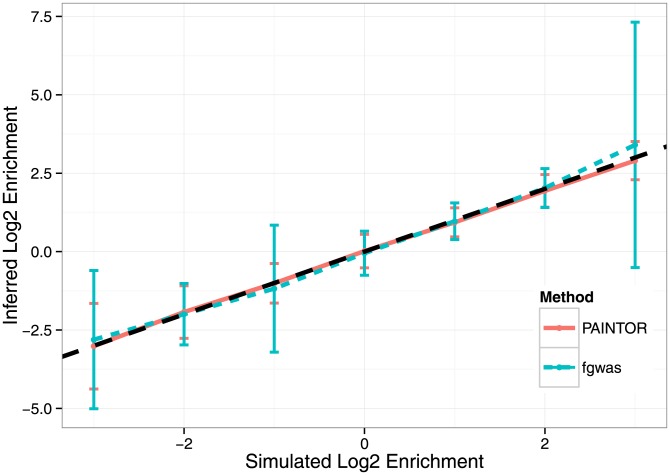
Accuracy of enrichment estimation for a synthetic annotation that contains 8-fold depletion to 8-fold enrichment of causal variants across simulations of fine-mapping data sets over 100 loci. Using a background and a synthetic functional annotation at a frequency of 1/3 (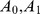), we simulated with annotation effect sizes such that in expectation, we attained approximately 100 causal variants while maintaining enrichment at a fixed point. We used the standard simulation parameters, fixing the variance explained by these 100 loci to 0.25 and using 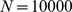 genotypes. We discarded simulations where fgwas failed to converge (see Methods). Displayed here are the mean inferred Log2 enrichment estimates (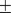 1 SD) that were conducted over 500 independent simulations at each enrichment level.

A vast resource for functional annotations is the ENCODE project [18], which has ascribed regulatory biological function to a large fraction of the human genome and has shown that regulatory DNA regions are highly cell-specific. Coupling this insight with the fact that for most complex diseases the relevant tissues are unknown, stresses the importance of carefully selecting cell-specific annotations for any specific trait [22]. A byproduct of our framework is the estimation of enrichment of causal variants within functional annotations (i.e. the ratio of prior probability of causality for SNPs within annotation versus those outside the annotation). Therefore, we can use PAINTOR to infer which functional annotations show significant effect on the probability of causality and use only those annotations to estimate probability of causality. To assess how accurately PAINTOR can recapitulate functional enrichment, we simulated fine-mapping studies over 100 loci with a synthetic functional annotation (see Methods) and either enriched or depleted causal variants within this annotation. We also compared our approach to fgwas [10] as it too is capable of inferring enrichment from summary data. Figure 3 demonstrates that both PAINTOR and fgwas are able to provide unbiased estimates of enrichment. However, we find that PAINTOR is more efficient than fgwas, and has a smaller variance attached to those estimates. We note that as causal variants become increasingly depleted from functional categories, fgwas tends to fail to converge (e.g. fgwas fails in nearly 21% of cases for simulations with 8-fold depletions). Finally, we assessed PAINTOR and fgwas for more realistic annotation data (i.e. contiguous segments in the genome) and find that both methods attain very similar results (see Figure S2).

### Selecting the optimal number of SNPs for functional testing

**Figure 4:**
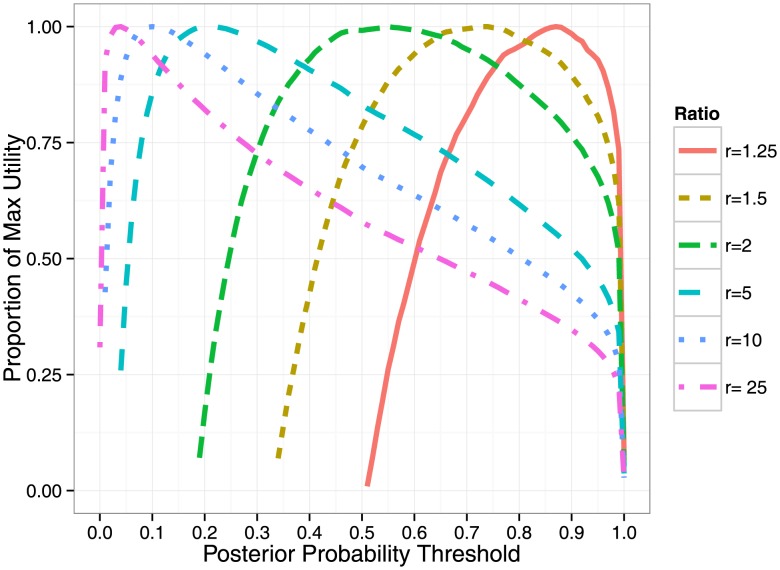
Thresholding on posterior probabilities provides a principled way to assess utility. We demonstrate how utility curves are optimized by selecting SNPs that achieve a minimum posterior probability threshold at various benefit-to-cost ratios (R). The total number of SNPs selected at the maximum utility for R =  (1.25, 1.5, 2, 5, 10, 20) is (29.8, 39.2, 52.4, 119.1, 221.4, 405.4) which identifies approximately (29.8, 35.6, 43.4, 65.33, 79.9, 91.8) causal variants.

Although PAINTOR (and previous methods) provide a quantification of the probability of each variant to be causal that can be used to rank variants based on their plausible causality, it remains unclear how to choose the number of variants to test in functional assays. The optimum number is constrained by the budget of the study and by an implicit cost to benefit ratio for selecting the optimal number of SNPs to be followed up. We propose a framework that assumes that every causal variant identified adds a benefit (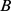) while every selected variant is tested at a cost (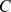); therefore, the utility function we propose to maximize is 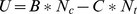, where 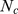 is the total number of true causal variants from the total number of selected SNPs (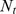). We note that the ratio 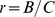 is the critical parameter of the utility function. Using the results from simulations with functional annotation enrichment described above, we assessed the capacity of the proposed utility function in selecting the number of SNPs for follow-up under various values for the ratio 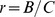 (Figure 4). For example, at a ratio 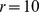 (the benefit of finding a causal outweighs 10 times the cost of testing 1 SNP), the utility is maximized by selecting approximately 3.5 SNPs per locus for validation resulting in 72.6% of causal variants successfully identified (see Figure S3).

Selection of a set of variants for follow-up is usually performed based on a threshold on posterior probability or based on credible sets that account for a given amount (e.g. 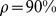) of the probability of capturing all causal variants [5], [8]. We assessed these two strategies for selecting variants for functional testing within the context of our benefit-to-cost framework. We find that a posterior probability threshold of (0.9, 0.5, 0.1) roughly corresponds to optimizing benefit-to-cost-ratios of 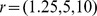. These results suggest that a simple translation of the arbitrary thresholds on posterior probabilities into cost-to-benefit optimum is attainable. In a similar fashion, we can assess credible sets within our cost-to-benefit framework. For example, the 90% credible set yields an average of 393 SNPs which is approximately 88% of the optimum for a benefit-to-cost of 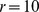.

### Application to meta-analysis data of lipid phenotypes

To validate our approach, we applied PAINTOR to association summary data from a large meta-analysis of four lipid traits. Our goal was to build a model that incorporated all the independent sources of available information (i.e. association signals alongside carefully selected functional annotations) to produce a prioritization of plausible causal SNPs for these phenotypes. We used the GWAS hits reported by Teslovich et al. [33] under the assumption that these regions contain causal variants and therefore well-suited to fine-map using PAINTOR. We first ran our method on 450 cell-type-specific annotations (see Methods) and fit the model to each annotation independently on both the original and densely imputed data sets for all four traits. Consistent to previous works, we observe that imputation consistently enhances the signal of enrichment [34], [23], [10]; for example, for HDL, the relative probability for causality for coding exons increases from 7.4 to 12.4 from using the original data to 1KG-imputed data (see Table S4). This effect is most likely due to the availability of more variants through imputation thus being able to localize the association signal to genomic annotation more accurately. Across the four traits in general, we see consistent signal of increased relative probability for causality within transcribed regions (e.g. exons and transcription start sites (TSS)) and a depletion of causal variants in repressed regions; for example, for TG, the coding exons show a log2 relative probability for causality of 3.4 while the repressed regions show an log2 relative probability of −1.6.

Having identified functional annotations that are relevant to the four traits of interest (see Table 5), we devised trait-specific PAINTOR models that included the top marginal annotations in conjunction with the association statistics to estimate the probability of causality for all SNPs from the risk loci on the densely imputed data sets (see Methods). Table 6 shows the HDL SNPs that attain a posterior PAINTOR probability greater than 0.9 (results for the other traits are displayed in the Tables S5, S6, S7). Unsurprisingly, the majority of these top SNPs localize in functional elements and attain a high marginal association statistic. We observe an abundance of liver associated cell types, DNase Hypersensitivity Sites, and genic elements annotated to these top SNPs. Notably, PAINTOR identifies four non-synonymous variants (rs7607980, rs1260326, rs5110, rs13107325), two of which were not reported in the initial Teslovich et al. findings. Overall by incorporating functional annotations we see a marked improvement in fine-mapping resolution across all four traits as indicated by a reduction in the 90% confidence sets relative to PAINTOR models with no annotations of 19.0%, 34.9%, 50.6%, and 24.2% for HDL, LDL, TC, and TG, respectively (Table 7). This corresponds to approximately an average reduction of 17.5 to 13.5 SNPs per locus across the four traits.

**Table 5:** Top 10 most significant annotations for lipid traits.

Phenotype	Cell Line	Type	Frequency	Log2 Relative Probability to be Causal	P.value
HDL	hepg2	TSS*	2.2%	3.46	1e-5
	-	Coding Exons*	1.4%	3.63	1e-3
	K562	Weak Enhancer*	0.7%	3.74	0.01
	MCF7	DHS*	30.5%	1.18	0.03
	gm12878	TSS*	1.8%	2.38	0.04
	fMuscle (leg)	DHS	39.5%	1.02	0.04
	fKidney (renal cortex)	DHS	26.9%	−1.57	0.04
	fStomach	DHS	33.9%	−1.14	0.07
	-	DHS UCSC	NA%	1.08	0.07
	h1hesc	Promoter Flanking	2.9%	1.84	0.08
LDL	fKidney	DHS*	40.4%	2.23	6e-3
	fLung	DHS*	34.3%	1.99	7e-3
	-	Coding Exons*	3.9%	2.92	0.01
	Hepatocytes	DHS*	38.2%	1.97	0.02
	HAsp	DHS*	33.7%	1.76	0.02
	WI_38	DHS *	31.9%	1.72	0.02
	AG09309	DHS	36.9%	1.68	0.02
	HFF_MyC	DHS	41.9%	1.61	0.02
	fKidney (renal cortex)	DHS	38.7%	1.73	0.03
	fKidney (renal pelvis)	rDHS	41.2%	1.67	0.03
TC	hepg2	Repressed*	53.9%	−1.87	6e-3
	fLung	DHS*	30.4%	2.18	6e-3
	fIntestine(Lg)	DHS*	18.8%	1.93	0.01
	hepg2	Transcribed*	31.2%	1.64	0.01
	NHDF_Neo	DHS*	26.0%	1.76	0.02
	k562	Repressed	60.9%	−1.51	0.03
	LNCap	DHS	41.9%	1.68	0.04
	fStomach	DHS	32.6%	1.50	0.04
	AoAF	DHS	27.9%	1.39	0.05
	fIntestine (Sm)	DHS	33.6%	1.39	0.06
	gm12878	Transcribed	26.3%	1.36	0.06
TG	-	Coding Exons*	1.5%	3.42	3e-3
	hepg2	Repressed*	57.9%	−1.63	6e-3
	GM19238	DHS*	22.4%	1.71	0.01
	fIntestine (Sm)	DHS*	29.9%	1.67	0.01
	-	Non-coding Exon*	2.5%	2.81	0.02
	-	DNASE UCSC	21.7%	1.72	0.02
	fKidney	DHS	31.9%	1.54	0.02
	fThymus	DHS	27.6%	1.39	0.04
	pHTE	DHS	39.5%	−1.52	0.04
	-	3′ UTR Exons	1.8%	2.97	0.04
	NT2	DHS	26.9%	−2.66	0.05

Displayed are the log2 relative probabilities of SNPs to be causal if they fall within the listed annotation. *Indicates use in final PAINTOR model for the phenotype.

**Table 6:** HDL SNPs with high confidence for causality.

rsID	Chrom	Pos	-Log10(P.value)	PAINTOR Probability	Annotations
rs1366544	chr16	56964719	43.86	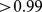	K562 Weak Enchancers, MCF7 DHS
rs1645788	chr19	54808174	10.79	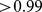	MCF7 DHS
rs3136447	chr11	46744368	16.08	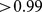	hepg2 TSS, MCF7 DHS
rs7241918	chr18	47160953	48.86	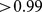	-
rs1077834	chr15	58723479	83.32	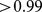	-
rs367070	chr19	54800500	14.69	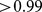	-
rs3809630	chr16	67879400	32.32	0.99	hepg2 TSS, MCF7 DHS, gm12878 TSS
rs7239867	chr18	47164717	47.53	0.99	-
rs13107325 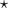	chr4	103188709	10.44	0.97	Coding Exons
rs7607980 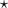	chr2	165551201	9.71	0.96	hepg2 TSS, Coding Exons, MCF7 DHS
rs4366775	chr17	76382079	8.50	0.93	gm12878 TSS
rs737337	chr19	11347493	8.81	0.92	hepg2 TSS, Coding Exons, MCF7 DHS
rs4490057	chr17	76375095	8.20	0.90	hepg2 TSS, MCF7 DHS, gm12878 TSS

SNPs with posterior probability causality 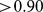 for HDL phenotype across the 37 risk loci (Results for TG/TC/LDL in Tables S5, S6, S7).

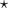
 denotes a non-synonymous variant.

**Table 7:** Reduction in the number of SNPs in the 90% Credible Set after incorporating functional annotations.

Phenotype	Total SNPs	SNPs with P-value <5e-8	Annotations	# SNPs	# Loci	# SNPS per locus
HDL	10778	1792	–	926	37	25.0
			+	778		21.0
LDL	3903	955	–	112	14	8
			+	83		5.9
TG	5513	975	–	488	23	20.3
			+	324		13.5
TC	5504	1381	–	390	24	17.0
			+	314		13.7
Average	6425	1276	–	479	24.5	17.5
			+	375		13.5

Shown here are the number of SNPs in the 90% Confidence Set for each of the lipid phenotypes as estimated using PAINTOR. After marginally running PAINTOR on the entire pool of annotations, we selected the top five annotations for each trait and fit full trait-specific models on each of the densely imputed data sets. We compared PAINTOR with or without integration of functional annotation data. The magnitude in the reduction in the size of the confidence set approximately mirrors what we observe in simulations.

## Discussion

Recent efforts by large consortia such the ENCODE have provided a genomic map of regulatory regions and have shown that GWAS associated variants are preferentially enriched within these regions. In this work, we propose a principled approach to unifying these genomic features with the standard association signal to improve the localization accuracy in fine-mapping studies. Our method relies on empirical data to select trait-specific genomic annotations, thus removing the need for ad-hoc selection of relevant functional annotations a priori. Through simulated and real data results, we have shown that our integrative framework is able to reduce the number of variants that need to be investigated to identify causal variants that alter risk of disease.

Our method shares similarities to recent integrative approaches proposed in the context of GWAS [10]. Although conceptually both approaches integrate functional and association signal, the two methodologies are fundamentally distinct in their aims. Whereas [10] seeks to identify novel risk loci by leveraging functional information, we instead propose our method as way to refine signal at known GWAS loci. This fundamental distinction leads to different statistical models and optimization procedures allowing for superior accuracy for refining association signal through fine-mapping. In addition our method addresses a limitation of [10] by allowing for the possibility of multiple causal variants at a risk locus.

Several hierarchical Bayesian methods have been developed that combine prior information with genomic association data to help prioritize variants in various contexts [29], [30]. The main contribution of our approach is that we explicitly account for LD between SNPs which we can learn from external reference panels such as the 1000 Genomes. Additionally, because we do not take a fully Bayesian approach [30] (i.e. integrate over the entire hyper-parameter space), we are able to devise computationally efficient algorithms that allow our method to search over the ever-increasing number of functional annotations (e.g. ENCODE) to identify the most informative subset while retaining the ability to model multiple causal variants.

We have shown that PAINTOR can unbiasedly estimate enrichment of causal variants in different functional elements on the basis of summary association data alone. This may prove to be particularly important as access to individual genotype data is more cumbersome than summary-level statistics. The unbiased nature of the estimation procedure may provide clues to the genetic basis of common traits. For example our results suggest that although coding variants are more likely to be causal than regulatory variants, the majority of the genetic variation contributing to the trait at these risk loci may lie within regulatory as opposed to coding regions due to the larger number of variants residing in regulatory regions. This is consistent with recent work that concluded that variants in regulatory regions show a higher contribution to traits than coding variants, however, such an analysis required individual level data [23].

One interesting implication of our results is that while higher-order functional data is very useful for gleaning insight into to the genetic architecture of human diseases genome-wide [20], [23], a critical component of accuracy in a fine-mapping study is the sample size (see Table S8). Consequently, the success of a fine-mapping experiment may hinge on first obtaining an adequate sample size and then augmenting that sample size with functional data. These findings are largely in-line with what was previously reported in the context of GWAS [10].

In this work we have applied our framework to known risk loci identified in GWAS in the search for plausible causal variants. As future work, our approach could be extended to risk loci that do not pass a genome-wide stringency, potentially leading to discovery of novel risk loci. Additionally, risk loci for related traits that are known to share a genetic basis could potentially be combined, leading to an increase in power to identify variants that contribute to both traits. Finally, we anticipate that the approximations of the non-centrality parameters could be handled in a more principled fashion using a Bayesian approach that integrates a prior distribution of effect sizes. We leave a thorough investigation of these directions as future work.

## Methods

### PAINTOR probabilistic model

A standard approach to model the strength of association of genotype to phenotype is through the Z-score. For a continuous phenotype, the trait values are marginally regressed on each SNP and the corresponding Z-score is taken to be the Wald statistic (i.e 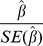), which is distributed 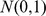 under the null. For case-control designs, the Z-score can also be obtained through the standard test statistic for two proportions (assuming equal sample sizes of 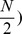: 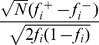, where 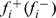 denotes the frequency of the SNP in the cases (controls) and 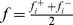. We define a fine-mapping locus as a contiguous region of the genome flanking a GWAS “hit” on both sides. Let 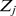 be a vector of Z-scores from the 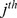 locus 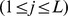 of length 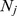. In addition, let 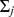 be the corresponding LD matrix of pairwise correlation coefficients for locus 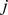 that can be derived directly from individual level data if available, or approximated using an appropriate reference panel such as the 1000 Genomes. We obtain 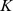 annotations 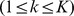 from external repositories (e.g. ENCODE [18]) and for each SNP 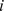, create a 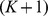-length binary annotation vector 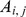, where 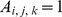 if the 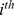 SNP at the 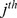 locus is part of annotation 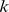. For example, one such annotation could be all coding sites and the annotation vector will contain a 1 only if the SNP is located within coding region. We note that 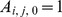 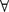 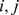 and serves to represent the “baseline” annotation whose corresponding coefficient can be interpreted as the baseline prior odds for causality of any SNP within the set of fine-mapping loci. Let 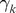 be the effect size of the 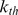 annotation on the probability of a SNP being causal and the non-centrality parameter, 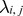, be the standardized effect size of SNP 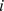 at locus 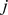. Finally, let 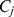 be an indicator vector of causality where 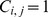 if SNP 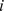 at locus 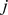 is causal and 0 otherwise. Now, we can define the likelihood of the data relative to these terms as:
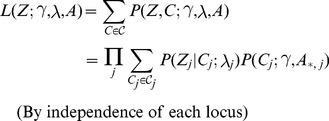
(1)where the sum is taken across all causal indicator vector sets 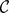. We note that in order to keep the enumeration of the causal vector sets combinatorially tractable, we restrict the total number of potential causal variants at each locus to three or less in practice (see Figure S4 for assessment of run time versus number of causal variants considered). We define the annotation effect on the causal probability through a standard logistic model:



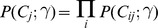


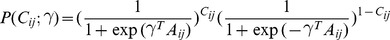
(2)and relate the causal set of SNPs to the observed association Z-scores under a standard multivariate normal assumption [35], [36], [37] as:
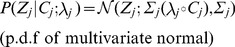
(3)where 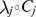 denotes the elemental pairwise multiplication between two vectors. A summary of model parameters can be found in Table 8.

**Table 8:** List of model parameters for the 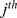 locus 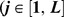 where L is the total number of fine-mapping loci).

Parameter	Description
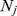	Number of SNPs at the the 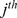 locus
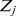	Vector of Z-scores (1× 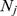 )
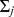	Linkage disequilibrium matrix consisting of pairwise Pearson Correlation coefficients between SNPs ( 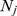 × 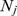 )
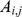	Vector of annotations for the 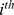 SNP. 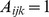 if member of annotation (1× 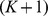 )
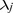	Vector of Non-centrality parameters (NCPs) (1× 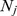 )
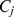	Indicator vector giving the causal status of all the SNPs at a locus. 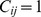 if the 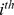 SNP is causal (1× 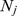 ).
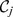	Set of all possible causal configurations. ( 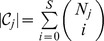 , where 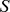 = number of causals one wants to consider at a locus).

### Model fitting

In order to compute the probability of causality, we must first fit the data to our model. We accomplish this through a maximum likelihood estimation over 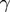. The formulation of our approach lends itself to the standard Expectation Maximization (EM) algorithm. The E-step of the EM involves computing at each locus independently, the posterior probability of each 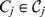 using an application of Bayes Theorem:
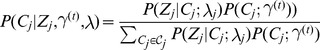
(4)


To obtain the posterior probability, 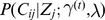, for each SNP*_i,j_* we marginalize across all 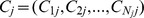 such that 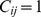.
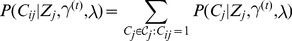
(5)


Despite the fact that posterior probabilities are calculated independently at each locus, we can set up the objective function to aggregate the results and borrow information across loci to compute estimates of 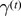. In doing so, we prevent over fitting of the data to any one locus, offering more robust estimates of the model parameters leading, in turn, to more accurate posterior probabilities. We define our Q function for the M step as follows
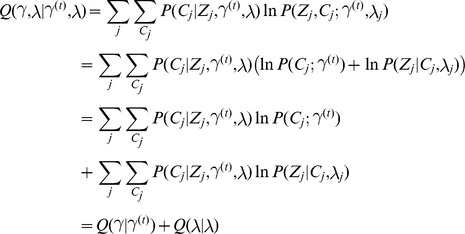



thereby partitioning the likelihood, decoupling the estimation of 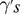 from the 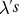. We simplify 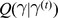 to obtain
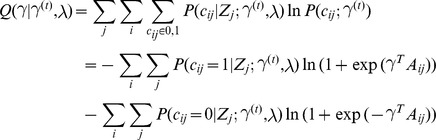
which is a concave function whose gradient is simply



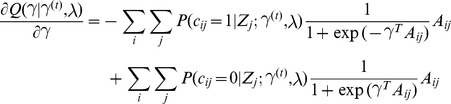



We optimized this function using the NLopt C++ package's implementation of the limited-memory BFGS algorithm [38], a quasi-Newton method that only requires the objective and the gradient as input [39]. As stated previously, we fix the non-centrality parameters, 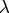, and only optimize over 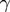 due to the fact that our model would be over-specified otherwise. Specifically, we set the non-centrality parameters at each SNP to the observed Z-score if the absolute Z-score is greater than 3.7 (corresponding to a p-value of 10e-4) or the sign of the observed Z-score times 3.7 otherwise. Simulation results show that our strategy yields high accuracy to detect causal variants among several simulated approaches to approximate 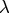 (Figure S5).

### Simulation framework

Starting from the 1000 Genomes (1 KG) European samples, we used HAPGEN [40] to simulate fine-mapping data sets over 10 Kb loci. We filtered monomorphic/rare SNPs (MAF 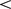 0.01) and normalized genotypes to be mean-centered with unit variance. For each simulation we randomly chose one hundred 10 Kb loci and randomly assigned SNPs to binary annotations at a pre-specified proportion. We drew causal status for each SNP according to the logistic model above and varied 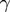 to induce a desired prior probability for causality for SNPs part of the “functional” annotation, while maintaining an approximately fixed number of causals – typically one per locus in expectation. For example, to induce an 8-fold causal enrichment in a synthetic “functional” annotation that contained 1/3 of the SNPs, the (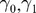) values were set to be (4.62, −2.15). We note that the random assignment of causal status would lead to loci with either zero (36), one (34), or multiple causal (30) variants on the average.

Once we established the causal SNPs, we used a linear model to simulate continuous phenotypes such that the causal SNPs aggregated to explain a fixed proportion of the phenotypic variance (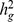). This phenotypic variance was partitioned equally amongst all the causal SNPs (qualitatively similar results were obtained when phenotypic variance was unevenly partitioned among causal variants (see Figure S6)). In particular, the 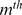 individual's phenotype was drawn according to 
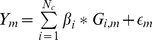
, where 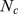 is the total number of causal variants, 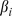 is the effect size of the 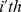 causal SNP, 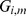 is number of copies of the risk allele 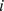 (randomly assigned as reference or alternate) for individual m, and 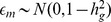. Finally, we calculated association Z-scores (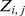) at each SNP 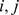 by taking the Wald statistic from the regression of the 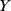 on 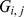, where Y is a vector of phenotypes for M individuals and 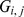 is the vector of corresponding genotypes for the 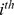 SNP at the 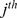 locus. For simulations that required loci greater than 10 KB, we instead drew Z-scores from a Multivariate Normal distribution with covariance equal to LD based on the European 1 KG and non-centrality parameters at causal sites drawn from a Normal distribution with mean 5 and standard deviation 0.2. When measuring performance of our simulations, we examine the proportion of causal SNPs identified as a function of the average number of SNPs per locus selected for follow-up restricted to loci that contain at least one causal variant (we show in Figure S7 that using Positive Predictive Value as a metric of accuracy attains qualitatively similar results).

### Existing approaches for fine mapping

We compared our approach to a several of existing methods that can be used for fine-mapping[5], [10], [12], [13], [14]. To compute Maller et al. [5] posterior probabilities, we first calculated Bayes factors with the R package, BayesFactor, using the default settings. We converted the resultant Bayes factors into posterior probabilities of association using the following formula: 
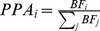
. We show in Figure S8 and Supplementary Note S1 that posterior probabilities approximated directly from the Z-scores give virtually indistinguishable results. We downloaded fgwas [10] version 0.3.4 from GitHub and ran the software using the -*fine* flag. Due to the fact that we fit linear models to obtain Wald statistics for each SNP, we were able to provide standard errors for the estimates of the prior variance. We segmented our input based on the individual loci as instructed in user manual, but provided a single file that contained all the fine-mapping SNPs so that fgwas could compute enrichment. The Guan and Stephens [14] method is implemented in the software *piMass* which we ran using the flags and MCMC parameters given in the user manual as defaults (burn-in  = 1000, samples  = 100,000, step-size  = 10). We used the posterior inclusion probabilities (PIPs) that had undergone Rao-Blackwellisation for prioritization due to the fact these had superior performance over naive PIPs. The R package implementing LLARRMA [13] was run using the default settings. Zuber et al. was implemented in the R package, care, which we also applied to the data using the default settings. We prioritized variants using the square of the CAT scores as described in [12]. We note that with exception fgwas, all the aforementioned methods were applied to each locus independently. Conditional analysis is a common procedure used to tease out secondary signals at associated loci [41]. For a single locus, we iteratively condition on the SNP most strongly associated with the simulated phenotype. We accomplish this in a step-wise fashion through marginal regression of the phenotype onto each SNP and subsequently conditioning on the one that is most significantly associated. At each iteration a new SNP will enter the regression model as a covariate until all the causal SNPs have been selected. The order in which the SNPs enter the model provides a natural ranking thus enabling us to compare iterative conditioning to other methods that rank SNPs probabilistically. As expected, we show in Figure S1 that conditioning is suboptimal for fine-mapping.

### Functional information

We explored whether integration of the location of tissue-specific regulatory and coding DNA regions could increase resolution in statistical fine-mapping. The ENCODE [18] project provided a wealth of genomic landmarks that were systematically integrated to segment the genome into seven major classes: transcription start site and predicted promotor region (TSS) (1.2%), predicted promotor flanking region (PF) (0.7%) predicted enhancer (E) (1.8%) predicted weak enhancer (WE) (2.5%), CTCF-enriched element (CTCF) (0.1%) predicted transcribed region (T) (19.3%) and finally, predicted repressed or low-activity region (R) (69.6%). We examined these genomic segmentations for the six primary ENCODE cell lines: gm12878 (lymphoblastoid), h1hesc (embryonic stem cells), helas3 (cervical cancer), hepg2 (liver carcinoma), huvec(umbilical vein endothelial cells), and k562 (chronic myelogenous leukemia). In addition, we also explored 403 broadly defined (peak 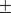 1 Kb) DNase I Hypersensitivity Sites spanning numerous tissues and cell lines. Of these 403 DHS I maps, 349 came from Maurano et al. [19], 73 DHS I annotations from Thurman et al. [42], with the remaining DHS annotation being an overall DHS map derived from UCSC genome browser. These annotations have been used recently in the context of GWAS [10].

### Measuring enrichment significance

Due to the fact that we fit our model using maximum likelihood, a natural way to ascribe statistical confidence to the inferred parameters is to use a likelihood ratio test. For example, to calculate the significance of a single annotation, we can compare a fitted null model to a model that contains the annotation under consideration using the following test statistic: 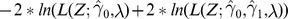. We demonstrate in simulations that under the null, this test statistic follows approximately its theoretical 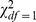 distribution (see Figure S9).

In addition to a point estimate for the enrichment of functional annotation, it would be useful to derive an estimate of the variance. Unfortunately, the complex structure of the likelihood makes it difficult to derive an analytically tractable parameter covariance estimator. However, since we assume fine-mapping loci to be independent, we propose to use bootstrapping (i.e. re-sampling entire loci with replacement) and subsequently re-fitting the model (see Methods). We confirm that such a strategy does indeed reproduce a correct estimate of the parameter variance in simulations. We find that the mean bootstrap standard deviation largely mirrors the “true” standard deviation of the parameter estimates (see Figure S10). As a result, a confidence interval based on the bootstrap standard deviation will attain desirable coverage properties due to the fact that estimation of the model parameters is unbiased.

### An optimization framework for selecting the number of SNPs to follow-up

The budget of a fine mapping follow-up study constrains the total number of causal variants to be further examined for evidence of causality. This motivates approaches that, in addition to providing a prioritization of SNPs, also identify an optimal number of SNPs to be tested. We introduce here a benefit-to-cost framework for selecting the optimal number of SNPs for follow-up. Our framework assumes that every causal variant identified adds a benefit (B) while every selected variant is tested at a cost (C); therefore, the utility function we propose to maximize is U = B*Nc - C*Nt, where Nc is the total number of true causal variants identified from the total number of selected SNPs. A key parameter of the utility framework is the ratio of 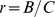 of benefit to utility.

### Lipids data set

Publicly available GWAS summary data across four blood lipids phenotypes was downloaded from public access websites [43]. Data was part of a meta-analysis conducted in 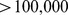 individuals of European ancestry that examined four plasma lipid traits (number of significant loci): LDL cholesterol (14 loci), HDL cholesterol (37 loci), trigylcerides (23 loci), and total cholesterol (24 loci). From the original 2.0 M SNPs, we imputed an additional 5.3 million Z-scores using ImpG-Summary [34]. For each significant GWAS hit reported by Teslovich et al., we centered a 100 KB window on the lead SNP and estimated LD from the European reference panel of the 1 KG. We chose a conservative window of 50 Kb on either side of the GWAS hit, as it has been previously shown that within European populations, average LD decays after 25 KB [44]. These loci contained an average of 718 SNPs in the 1000 genomes reference panel, of which we were able to on average accurately impute 261 (see Table S9). This resulted in 2837 (10778), 1231 (3903), 1693 (5504), 1615 (5513) SNPs (with 1 KG imputation) to which we fit our model for HDL, LDL, TC, and TG respectively. In addition, we created the corresponding pool of functional annotations described above for every SNP in a window. We analyzed the dataset using PAINTOR in two phases. In the first phase we fit our model for each annotation independently to ascertain the functional annotations most phenotypically relevant. We did this for all four lipid traits for both the original and densely imputed data sets. After running PAINTOR marginally on each annotation, we selected the the top five most significant annotations for the final model (denoted with a * in Table: 5). We note that in the case of experimental replicates (i.e. same tissue and class), we only report the top replicate.

## Supporting Information

Figure S1 Single locus fine-mapping using four different prioritization strategies. Using HAPGEN-derived genotypes from a randomly selected a 10KB locus on chromosome 1, we simulated 10,000 fine-mapping data over N = 2500 samples at a locus that explains 5% of variance in the phenotype. Each variant has a prior probability of 1/L (where L is the total number of variants at the locus) to be casual; the total variance was divided equally among variants when multiple causal variants were present. As previously observed, prioritization under the assumption of a single causal variant is identical to ranking based on p values at a single locus.
(EPS)

Figure S2Contiguous annotations do not lead to appreciably different performance to randomly assigned annotations. Displayed here is the accuracy of enrichment estimation for a synthetic annotation that contains 8-fold depletion to 8-fold enrichment of causal variants across simulations of fine-mapping data sets over 100 loci. We enriched causal variants in an annotation that spanned a block 1/3 of the size of the locus and simulated with annotation effect sizes such that in expectation, we attained approximately 100 causal variants while maintaining enrichment at a fixed point. We used the standard simulation parameters, fixing the variance explained by these 100 loci to 0.25 and using 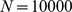 genotypes. We discarded simulations where fgwas fails to converge (see Methods).(EPS)

Figure S3Selection of optimal fine-mapping set according to an utility function. Using our standard simulation parameters (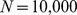 and 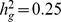), causal variants were enriched in three functional annotations at relative marginal probabilities of 9.5, 5.7, and 3.65. Since different ratios will give different scales for the utility function, we normalize the output by the maximum utility.(EPS)

Figure S4Runtime scales exponentially as the number of causal variants integrated over increases. We assesed run-time within the context of our standard simulation framework (ten simulations per point) and varied the number of causal variants PAINTOR integrated over. These simulations were carried out on a single core of an Intel(R) Xeon(R) E5335 2.00 GHz CPU. As the results suggest, we are required in practice to restrict the number of causal variants to a small fixed constant 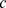 in order to keep the computational burden reasonable.(EPS)

Figure S5Performance using different strategies for approximating the non-centrality parameters 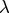. Observed Z-score corresponds to setting the 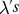 to the observed z-score at that SNP. Maximum z-score corresponds to setting the NCPs to the maximum z-score at the locus times the sign of the observed z-score. Standard NCP's is the strategy described in the main Methods section wherein the NCP's are set to to the observed Z-score if the absolute Z-score is greater than 3.7 (corresponding to a p-value of 10e-4) or the sign of the observed Z-score times 3.7 otherwise.(EPS)

Figure S6Overall performance with heterogenous SNPs effect sizes. To induce heterogeneity on SNPs, effect sizes of causal sites were drawn from an 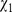. These effect sizes were then normalized such that their aggregated effect summed up to a heritability of 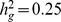. Other simulation parameters were equivalent to the standard framework (N = 10,000, Loci  = 100).(EPS)

Figure S7Comparison of current methodologies using positive predictive value (PPV) as the metric (defined as: 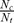). We find that the relative performance of all the methods investigated in this manuscript is maintained when assessing accuracy with the PPV.(EPS)

Figure S8Posterior probabilities for causality under the assumption of a single causal variant approximated from z-scores give indistinguishable performance to that of the Bayesian approach described in Maller et al. [5]. Using the standard simulation framework (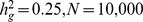) we calculated posterior probabilities from either Bayes Factors computed using the R package BayesFactor or directly from the association statistics. We then used these posterior probabilities to rank SNPs across all causal loci. The average tau rank correlation between the resulting posterior probabilities is 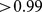.(EPS)

Figure S9QQ Plot of likelihood ratio test statistics for a single annotation. Using the standard simulation conditions (see Methods), we ran 5000 null simulations wherein 1/3 of the SNPs were annotated to a “functional” annotation with zero effect size. We calculated LRT statistics (see Methods) from each simulation which are theoretically distributed 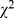 with df = 1 under the null. The resultant LRT statistics from the 5000 simulations have mean  = 1.005, variance  = 2.11, and median  = 0.44, suggesting that our test statistic is well-calibrated.(EPS)

Figure S10Bootstrap standard deviations for different log2 enrichment values. Using the standard simulation conditions (see Methods), we ran 100 simulations at three causal variant log2 enrichment values (−3,0,3) and for each of the simulations calculated 1000 bootstrap estimates. The standard deviations of the estimated 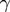 coefficients were calculated across the 100 simulations (blue) and compared to the mean standard deviations of the bootstrap estimates(red). Background and functional refer to the whether the annotation represents the background SNPs or the synthetic functional annotation that we randomly assigned to 1/3 of the SNPs.(EPS)

Table S1 Basic summary of fine-mapping methods assessed. We highlight the key contribution of our approach is that we can use PAINTOR to do fine-mapping with functional priors while modeling multiple causal variants directly from summary association statistics (Z-scores).
(PDF)

Table S2 Incorporating prior probabilities provides larger benefit when Z-scores at the causal SNPs are smaller. Here, we illustrate the efficacy of fine-mapping at loci where the p-value at the causal SNPs fall in either the top or bottom quartile of significance (as indicated by the absolute z-score).
(PDF)

Table S3 Performance of PAINTOR with and without integrating annotations if thresholding on the posterior probability (Average number of causals per simulation  = 108). The objective function is given as ratio from the maximum objective at a cost to benefit ratio of 10.
(PDF)

Table S4 Imputation boosts estimates of enrichment/depletion. The original data set was imputed up to the HapMap. Using ImpG-Summary we further imputed Z-scores up to the 1000 genomes reference panel. We combined enrichment estimates across all 4 phenotypes and examined the tails of log2 enrichment distributions.
(PDF)

Table S5LDL SNPs attaining PAINTOR posterior probabiliites 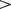
0.9 with functional annotations.(PDF)

Table S6TC SNPs attaining PAINTOR posterior probabiliites 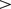
0.9 with functional annotations.(PDF)

Table S7TG SNPs attaining PAINTOR posterior probabiliites 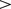
0.9 with functional annotations.(PDF)

Table S8Performance of PAINTOR as a function of sample size. We fixed the proportion of phenotypic variance explained in a simulated trait to 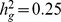 and selected a variable number of individuals to conduct fine-mapping experiments over. Displayed are the average number of SNPs per locus that need to be selected in order to identify the listed percentage of causals.(PDF)

Table S9Average number of SNPs that were well-imputed at the loci for the four lipid phenotypes. The top row corresponds to the average number of common SNPs in the 1000 Genomes reference panel at these loci. The bottom row corresponds to the average number of SNPs that were imputed with accuracy 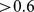 at these loci.(PDF)

Note S1 Comparison of methods for single locus fine-mapping and approximation of posterior probabilities from z-scores under the assumption of a single causal variant.
(PDF)
