# Supplementary material for: Integrating Functional Data to Prioritize Causal Variants in Statistical Fine-Mapping Studies
Source: PLoS Genet. 2014 Oct 30;10(10):e1004722. doi: 10.1371/journal.pgen.1004722 (PMC4214605; doi:10.1371/journal.pgen.1004722)
Supplement: Table S1 — Basic summary of fine-mapping methods assessed. We highlight the key contribution of our approach is that we can use PAINTOR to do fine-mapping with functional priors while modeling multiple causal variants directly from summary association statistics (Z-scores). (PDF) [file pgen.1004722.s011.pdf]

Method	Operate on Summary Data	Integrates Functional Priors	Can Handle Multiple Causal Variants
PAINTOR	Yes	Yes	Yes
Maller et al.	Yes	No	No
fgwas	Yes	Yes	No
piMass	No	No	Yes
LLARRMA	No	No	Yes
CAT score	No	No	Yes
