# Supplementary material for: Integrating Functional Data to Prioritize Causal Variants in Statistical Fine-Mapping Studies
Source: PLoS Genet. 2014 Oct 30;10(10):e1004722. doi: 10.1371/journal.pgen.1004722 (PMC4214605; doi:10.1371/journal.pgen.1004722)
Supplement: Table S2 — Incorporating prior probabilities provides larger benefit when Z-scores at the causal SNPs are smaller. Here, we illustrate the efficacy of fine-mapping at loci where the p-value at the causal SNPs fall in either the top or bottom quartile of significance (as indicated by the absolute z-score). (PDF) [file pgen.1004722.s012.pdf]

Significance Quartile	Fraction of Causals	PAINTOR	PAINTOR No Annot	Percent Change
Bottom 25%	0.50	17.98	26.37	31.79%
	0.90	130.32	165.83	21.41%
Top 25%	0.50	34.62	50.28	31.13%
	0.90	236.31	252.64	6.46%
