# Supplementary material for: Integrating Functional Data to Prioritize Causal Variants in Statistical Fine-Mapping Studies
Source: PLoS Genet. 2014 Oct 30;10(10):e1004722. doi: 10.1371/journal.pgen.1004722 (PMC4214605; doi:10.1371/journal.pgen.1004722)
Supplement: Table S3 — Performance of PAINTOR with and without integrating annotations if thresholding on the posterior probability (Average number of causals per simulation = 108). The objective function is given as ratio from the maximum objective at a cost to benefit ratio of 10. (PDF) [file pgen.1004722.s013.pdf]

Threshold on Posterior	PAINTOR			PAINTOR No Annotation		
	Total SNPs	Causals	Objective	TotalSNPs	Causals	Objective
0.10	232.85	78.61	1.00	265.16	73.79	1.00
0.20	132.59	65.52	0.93	128.20	56.09	0.90
0.30	93.51	57.09	0.84	80.28	45.68	0.77
0.40	72.61	50.84	0.76	57.92	39.20	0.68
0.50	57.96	45.00	0.68	43.03	33.23	0.59
0.60	48.78	40.54	0.62	35.88	30.06	0.53
0.70	41.33	36.30	0.56	31.23	27.59	0.48
0.80	34.20	31.60	0.48	27.16	25.17	0.44
0.90	27.56	26.51	0.40	22.95	22.07	0.38
