# Supplementary material for: Integrating Functional Data to Prioritize Causal Variants in Statistical Fine-Mapping Studies
Source: PLoS Genet. 2014 Oct 30;10(10):e1004722. doi: 10.1371/journal.pgen.1004722 (PMC4214605; doi:10.1371/journal.pgen.1004722)
Supplement: Table S4 — Imputation boosts estimates of enrichment/depletion. The original data set was imputed up to the HapMap. Using ImpG-Summary we further imputed Z-scores up to the 1000 genomes reference panel. We combined enrichment estimates across all 4 phenotypes and examined the tails of log2 enrichment distributions. (PDF) [file pgen.1004722.s014.pdf]

Percentile	HapMap	1000 Genomes
$\leq 10^{th}$	-8.73	-9.49
$\geq 90^{th}$	1.90	1.97
