# Supplementary material for: Integrating Functional Data to Prioritize Causal Variants in Statistical Fine-Mapping Studies
Source: PLoS Genet. 2014 Oct 30;10(10):e1004722. doi: 10.1371/journal.pgen.1004722 (PMC4214605; doi:10.1371/journal.pgen.1004722)
Supplement: Table S5 — LDL SNPs attaining PAINTOR posterior probabiliites 0.9 with functional annotations. (PDF) [file pgen.1004722.s015.pdf]

rsID	Chrom	Pos	-Log10(P.value)	PAINTOR Probability	Annotations
rs41290120	chr19	45382675	181.02	1.00	fKidney DHS, fLung DHS, Hepatocytes DHS, HAsp DHS
rs4420638	chr19	45422946	146.36	1.00	Hepatocytes DHS
rs5930	chr19	11224265	32.88	1.00	fKidney DHS, Coding Exons
rs4953023	chr2	44074000	32.78	1.00	fKidney DHS, fLung DHS, Hepatocytes DHS
rs6511720	chr19	11202306	116.67	1.00	fKidney DHS , fLung DHS, Hepatocytes DHS, HAsp DHS
rs7746081	chr6	16126934	13.26	0.98	fKidney DHS, fLung DHS, Hepatocytes DHS, HAsp DHS
rs629301	chr1	109818306	170.32	0.94	fKidney DHS ,fLung DHS, Hepatocytes DHS, HAsp DHS
rs1564348	chr6	160578860	17.07	0.93	fLung DHS ,HAsp DHS
