# Supplementary material for: Integrating Functional Data to Prioritize Causal Variants in Statistical Fine-Mapping Studies
Source: PLoS Genet. 2014 Oct 30;10(10):e1004722. doi: 10.1371/journal.pgen.1004722 (PMC4214605; doi:10.1371/journal.pgen.1004722)
Supplement: Table S6 — TC SNPs attaining PAINTOR posterior probabiliites 0.9 with functional annotations. (PDF) [file pgen.1004722.s016.pdf]

rsID	Chrom	Pos	-Log10(P.value)	PAINTOR Probability	Annotations
rs34006994	chr1	25780668	9.51	1.00	hepg2 Transcribed
rs2000999	chr16	72108093	23.79	1.00	fLung DHS, fIntestine(Lg) DHS, hepg2 Transcribed, NHDF_neo DHS
rs12916	chr5	74656539	46.35	0.97	fLung DHS, hepg2 Transcribed
rs6882076	chr5	156390297	27.43	0.95	hepg2 Repressed
rs7570971	chr2	135837906	8.02	0.95	hepg2 Transcribed
