# Supplementary material for: Integrating Functional Data to Prioritize Causal Variants in Statistical Fine-Mapping Studies
Source: PLoS Genet. 2014 Oct 30;10(10):e1004722. doi: 10.1371/journal.pgen.1004722 (PMC4214605; doi:10.1371/journal.pgen.1004722)
Supplement: Table S7 — TG SNPs attaining PAINTOR posterior probabiliites 0.9 with functional annotations. (PDF) [file pgen.1004722.s017.pdf]

rsID	Chrom	Pos	-Log10(P.value)	PAINTOR Probability	Annotations
rs1260326 ⋆	chr2	27730940	132.55	1.00	Coding Exons ,Non-coding Exons
rs138022915	chr8	19885934	98.98	1.00	hepg2 Repressed
rs138570705	chr15	44266730	-28.19	1.00	hepg2 Repressed , GM19238 DHS
rs4665985	chr2	27753878	52.44	1.00	hepg2 Repressed
rs5110⋆	chr11	116691634	34.09	1.00	Coding Exons , hepg2 Repressed, GM19238 DHS ,fIntestine(Sm)
rs964184	chr11	116648917	227.68	1.00	fIntestine(Sm)
rs114366307	chr8	19885726	98.98	1.00	hepg2 Repressed
rs11743303	chr5	55859952	9.13	1.00	hepg2 Repressed , GM19238 DHS, fIntestine(Sm)
rs2412710	chr15	42683787	8.03	0.99	GM19238 DHS , fIntestine(Sm)
