# Supplementary material for: Integrating Functional Data to Prioritize Causal Variants in Statistical Fine-Mapping Studies
Source: PLoS Genet. 2014 Oct 30;10(10):e1004722. doi: 10.1371/journal.pgen.1004722 (PMC4214605; doi:10.1371/journal.pgen.1004722)
Supplement: Table S8 — Performance of PAINTOR as a function of sample size. We fixed the proportion of phenotypic variance explained in a simulated trait to and selected a variable number of individuals to conduct fine-mapping experiments over. Displayed are the average number of SNPs per locus that need to be selected in order to identify the listed percentage of causals. (PDF) [file pgen.1004722.s018.pdf]

% Causal	N=1000	N=2500	N=5000	N=10000
10%	0.66	0.25	0.17	0.16
50%	8.0	4.2	2.28	1.6
90%	25.9	19.0	12.5	10.8
