# Supplementary material for: Integrating Functional Data to Prioritize Causal Variants in Statistical Fine-Mapping Studies
Source: PLoS Genet. 2014 Oct 30;10(10):e1004722. doi: 10.1371/journal.pgen.1004722 (PMC4214605; doi:10.1371/journal.pgen.1004722)
Supplement: Table S9 — Average number of SNPs that were well-imputed at the loci for the four lipid phenotypes. The top row corresponds to the average number of common SNPs in the 1000 Genomes reference panel at these loci. The bottom row corresponds to the average number of SNPs that were imputed with accuracy at these loci. (PDF) [file pgen.1004722.s019.pdf]

	HDL	LDL	TC	TG
1KG SNPS	766.81	736.71	677.75	694.57
Well-imputed	292.30	279.79	230.33	240.70
