# Supplementary material for: Integrating Functional Data to Prioritize Causal Variants in Statistical Fine-Mapping Studies
Source: PLoS Genet. 2014 Oct 30;10(10):e1004722. doi: 10.1371/journal.pgen.1004722 (PMC4214605; doi:10.1371/journal.pgen.1004722)
Supplement: Note S1 — Comparison of methods for single locus fine-mapping and approximation of posterior probabilities from z-scores under the assumption of a single causal variant. (PDF) [file pgen.1004722.s020.pdf]

Supplementary Note: Integrating functional data to prioritize causal variants in statistical fine-mapping studies

Gleb Kichaev[1], Wen-Yun Yang[2], Sara Lindstrom[3], Farhad Hormozdiari[2], Eleazar Eskin[1,2,4], Alkes L Price[3,5], Peter Kraft[3,5], and Bogdan Pasaniuc[1,4,6]

[1]Bioinformatics Inter-departamental Program, University of California Los Angeles, Los Angeles, CA., USA
[2]Dept of Computer Science, University of California Los Angeles, Los Angeles, CA., USA
[3]Program in Genetic Epidemiology and Statistical Genetics, Harvard School of Public Health, Boston, MA., USA.
[4]Dept of Human Genetics, David Geffen School of Medicine, University of California Los Angeles, Los Angeles, CA., USA
[5]Dept of Biostatistics, Harvard School of Public Health, Boston, MA., USA.
[6]Dept of Pathology and Laboratory Medicine,David Geffen School of Medicine University of California Los Angeles, Los Angeles, CA., USA

Supplementary Note

Single locus fine mapping

We compared various approaches for prioritizing variants for follow-up testing in fine-mapping at a single locus. Starting from 1000 Genomes European haplotypes, we used HAPGEN[1] to simulate fine-mapping data-sets over 2,500 individuals at a sequenced locus that explains $h^2 = 0.05$ of the variance in the phenotype (see Methods). PAINTOR attains superior performance over other methods (Figure S1). In particular, to identify (10%, 50%, 90%) of the total simulated causal variants, one needs to test (0.5, 3.3, 17.8) of SNPs if using PAINTOR as opposed to (0.6, 4.1, 22.7) SNPs for the Maller et al[2] approach or (0.7, 7.5, 34) if selected based on iterative conditioning. The increase in performance arises from modeling of multiple causal variants in our framework without losing power in simulations with one causal at the locus. Interestingly, the iterative conditioning approach attains good performance when selecting a small number of SNPs for followup and rapidly deteriorates as more variants are selected for follow-up; this is likely due to the fact that in the presence of strong LD between causal and tag variant, the conditional approach may completely miss the true causal variant if it first selects a tag SNP rather than the correlated causal SNP. This suggests that although conditional analysis may be effective in detecting secondary signal, it is not effective in discriminating the true causal variants in the presence of strong LD.

Estimating posterior probabilities from z-scores under the assumption of a single causal variant at the locus

We assume that the multi-variate vector of z-scores ($\bar{z}$) at a locus is distributed as a multi-variate normal with variance-covariance matrix induced by Σ (Σ contains all pairwise correlations among variants at the locus). We show next that the approach of using multi-variate distribution to account for LD is equivalent to using single-variate distribution when the causal SNPs have been typed. Let L_{ij} be the log likelihood

ratio of the SNP i to j. It follows that

$$
\begin{aligned}
L_{ij} &= logP(s_i|\bar{z}) - logP(s_j|\bar{z}) \\
&= logP(\bar{z}|s_i) - logP(\bar{z}|s_j) \\
&= -\frac{1}{2}[(\bar{z} - \Sigma_i\lambda_i)'\Sigma^{-1}(\bar{z} - \Sigma_i\lambda_i) + (\bar{z} - \Sigma_j\lambda_j)'\Sigma^{-1}(\bar{z} - \Sigma_j\lambda_j]
\end{aligned} \tag{1}
$$

where Σ_i is the i-th column of the variance covariance matrix Σ and λ_i is the effect size of SNP i. By setting λ_i as z_i it follows:

$$
\begin{aligned}
L_{ij} = &-\frac{1}{2}[\bar{z}'\Sigma^{-1}\bar{z} - \bar{z}'\Sigma^{-1}(\Sigma_i z_i) - (\Sigma_i z_i)'\Sigma^{-1}\bar{z} + (\Sigma_i z_i)'\Sigma^{-1}(\Sigma_i z_i) \\
&- \bar{z}'\Sigma^{-1}\bar{z} + \bar{z}'\Sigma^{-1}(\Sigma_j z_j) + (\Sigma_j z_j)'\Sigma^{-1}\bar{z} + (\Sigma_j z_j)'\Sigma^{-1}(\Sigma_j z_j)] \\
&= -\frac{1}{2}[-z_i^2 - z_i^2 + z_i^2 + z_j^2 + z^2 j - z_j^2] \\
&= -\frac{1}{2}[-z_i^2 + z_j^2] \\
&= \frac{z_i^2 - z_j^2}{2}
\end{aligned} \tag{2}
$$

which is equivalent to the log likelihood ratio under the univariate normal distribution of the marginal association statistics z_i and z_j. Given that the log likelihood ratio for any two SNPs is equivalent, it follows that under a single causal variant hypothesis with typed SNPs, the multi-variate and uni-variate normal frameworks are equivalent. Therefore,

$$
P(s_i|\bar{z}) = \frac{P(\bar{z}|s_i)P(s_i)}{P(\bar{z})} \tag{3}
$$

$$
\propto P(z_i|s_i)P(s_i) \tag{4}
$$

Assuming an uniform prior that any SNP i at a given locus is causal (i.e. $P(s_i) = 1/n$), we can calculate posterior probabilities of causality at a single locus as follows:

$$
P(s_i|\bar{z}) = \frac{Normal(z_i; 0, 1)}{\sum_j Normal(z_j; 0, 1)}
$$

We demonstrate in Figure S8 that calculating posterior probabilities using this approach gives similar performance to computing posterior probabilities of associations (PPA) using Bayes Factors[2].

References

[1] Su Z, Marchini J, Donnelly P (2011) Hapgen2: simulation of multiple disease snps. Bioinformatics .

[2] Maller JB, McVean G, Byrnes J, Vukcevic D, Palin K, et al. (2012) Bayesian refinement of association signals for 14 loci in 3 common diseases. Nature genetics 44: 1294–1301.
